# Modeling place-based nature-based solutions to promote urban carbon neutrality

**DOI:** 10.1007/s13280-023-01872-x

**Published:** 2023-05-15

**Authors:** Cong Cong, Haozhi Pan, Jessica Page, Stephan Barthel, Zahra Kalantari

**Affiliations:** 1 MIT Department of Urban Studies and Planning, Massachusetts Institute of Technology, Cambridge, MA USA; 2 Department of Urban and Regional Planning, University of Illinois at Urbana-Champaign, Champaign, IL USA; 3 School of International and Public Affairs, China Institute for Urban Governance, Shanghai Jiao Tong University, 1954 Huashan Rd., Shanghai, 200030 China; 4 Department of Physical Geography, Stockholm University, Stockholm, Sweden; 5 Department of Building Engineering, Energy Systems and Sustainability Science, University of Gävle, Gävle, Sweden; 6 Stockholm Resilience Centre, Stockholm University, Stockholm, Sweden; 7 Department of Sustainable Development, Environmental Science and Engineering (SEED), KTH Royal Institute of Technology, Stockholm, Sweden

**Keywords:** Carbon emissions, Carbon neutrality, Ecosystem services, Land use, Nature-based solutions

## Abstract

Nature-based solutions (NbS) are recognized as widely available and cost-effective mechanisms for sequestering carbon and offsetting carbon emissions. Realistic NbS implementations for carbon neutrality need to be effective at the global level and also appropriate for the socio-economic and physical conditions prevailing at the local level. This paper presents a framework that can help stakeholders identify demands, locations, and types of NbS interventions that could maximize NbS benefits at the local scale. Key processes in the framework include (1) interpolating carbon emissions data at larger spatial scales to high-resolution cells, using land use and socio-economic data; (2) assessing NbS effects on carbon reduction and their location-related suitability, through qualitative literature review, and (3) spatially allocating and coupling multiple NbS interventions to land use cells. The system was tested in Stockholm, Sweden. The findings show that the urban center should be allocated with combinations of improving access to green spaces and streetscapes, while the rural and suburban areas should prioritize preserving and utilizing natural areas. Our proposed method framework can help planners better select target locations for intended risk/hazard-mitigating interventions.

## Introduction

Urban areas contribute more than 60% of global greenhouse gas (GHG) emissions, through residential, commercial, and transportation activities (Lanau et al. 2021). Carbon sequestration by urban vegetation and natural amenities, such as parks, gardens, and residential lawns, can be a cost-effective mechanism for offsetting carbon emissions (Kalantari et al. 2019; Li and Wang 2021). Nature-based solutions (NbS) are increasingly being adopted by cities worldwide to enhance natural capital and preserve ecosystem service values (Lafortezza et al. 2018). NbS are defined by the European Commission as “solutions that are inspired and supported by nature, which are cost-effective, simultaneously provide environmental, social and economic benefits and help build resilience” (European Environment Agency 2021, p. 17).

A ‘toolbox’ developed for deploying NbS in urban settings are combinations of ecosystem service or green infrastructure approaches, including ecological restoration (Nesshöver et al. 2017; Dorst et al. 2019), soil-vegetation and landscape (Keesstra et al. 2018), biodiversity preservation (Kabisch et al. 2016), water management (Krauze and Wagner 2019; Gómez Martín et al. 2020), and integrated planning (Albert et al. 2019; Pan et al. 2021). Well-designed NbS can help mitigate carbon emissions and provide other social and economic co-benefits, including enhancing habitat quality, reducing the costs of artificial infrastructure, promoting participation and equitable access to amenities, creating jobs in the green sector, and improving recreational opportunities and health (Raymond et al. 2017; Giordano et al. 2020; Ruangpan et al. 2021).

Despite prior emphases on better NbS planning, a continuing absence of systematic approaches results in either (a) one-off and case-specific placement of infrastructure not entirely trusted by all stakeholders (Raymond et al. 2017); or (b) highly conceptualized models with low spatially detailed suitability (Connop et al. 2016; Kuller et al. 2019). Realistic NbS implementation plans toward carbon neutrality need to be both effective in mitigating carbon emissions at the global level and suitable for the socio-economic and physical conditions at the local level. Prioritizing suitable sites and solutions can enhance the long-term viability of NbS and its ability to provide multiple ecosystem services. To achieve this, it is necessary to fully consider the spatial details that are crucial for the successful implementation of NbS, including designing interventions that target specific emission sources. Fine-scale emissions analysis can help identify localized emissions driven by human behaviors, which may not be apparent when looking at emissions data on a larger scale.

In response to these challenges, this paper presents a novel systematic framework for prioritizing NbS types and locations in an integrative manner, to maximize their potential for mitigating carbon emissions. The method involves (1) fine-scale (30 × 30 m) spatial accounting and mapping of carbon emissions from transportation, residential, and industrial activities taking place within a study region; (2) a meta-analysis identifying relevant NbS strategies and their carbon emission reduction benefits in different places and cases, where NbS strategies include both direct carbon mitigation, such as carbon storage and sequestration, and indirect carbon mitigation, through climate regulation and interventions influencing human behaviors; and (3) prioritized NbS interventions allocated across the study region based on both their potential for carbon emission mitigation and their local suitability. Using the case of Stockholm County, Sweden, we tested the capacity of this framework for tailoring and targeting emission reduction approaches and visually communicating the carbon mitigation impacts of NbS strategies.

This paper addresses two main research questions: Which NbS strategies are most effective in mitigating urban carbon emissions? And where should selected types of NbS be deployed to maximize their carbon emissions saving potential? Acknowledging that the ability of NbS to mitigate carbon emissions goes beyond the carbon sequestration potential of green infrastructure and natural amenities, this paper focuses on the potential of NbS to offset human activity-related emissions including transportation, residential, and industrial emissions, and aims to provide a finer-scale assessment of the benefits of NbS.

The remainder of this paper is organized as follows: “Related works” section summarizes previous work on NbS and carbon emissions mitigation and identifies research gaps. “Materials and methods” section introduces our modeling methods, the case study area, and data sources. “Results” section presents the results obtained in the analysis and proposes an operational framework for place-based NbS. “Discussion” section discusses potential applications of the results and policy implications. “Conclusions” section presents some conclusions and discusses limitations of the study.

## Related works

### Adopting NbS for offsetting carbon emissions

Many studies have shown that urban NbS can provide multiple ecosystem services with environmental, social, and economic co-benefits, including climate change mitigation and adaptation (Chen 2015; De la Sota et al. 2019; Choi et al. 2021). For example, green infrastructure (GI) or green and blue infrastructure (GBI), as opposed to artificial (gray) infrastructure, can efficiently reduce urban carbon emissions. In a study by Anderson and Gough (2020), an average carbon dioxide (CO_2_) reduction of 6% was achieved by the application of GI in the built environment. According to Ren et al. (2019), the average annual increase in carbon storage by urban GI offsets 3.9% of the increase in urban carbon emissions in China. A study by Tomalty (2012) found that forestland, wetlands, and agricultural land in Ontario’s Greenbelt around Toronto can store 86.6 million tons of carbon and sequester 200 000 tons of carbon annually.

NbS can reduce carbon emission levels through different direct and indirect pathways. Direct pathways typically refer to the natural growth of vegetation, during which plants remove atmospheric CO_2_ and store it in their biomass (Nowak and Crane 2002). Several studies have quantified CO_2_ storage and sequestration by urban forests, e.g., Zhao et al. (2010) report carbon sequestration by urban forests in Hangzhou, China, of over 1.3 million metric tons (MMT) C/year, offsetting 18.6% of annual industrial carbon emissions with carbon storage equivalent to 1.75 times the annual industrial amount emitted. A later study by Chen (2015) estimated total carbon sequestration of 1.90 MMT from GI, with an average rate of 2.16 t/ha/year, in 35 major cities of China, offsetting up to 22.5% of carbon emissions from fossil fuel combustion.

NbS also reduce carbon emissions through indirect pathways, with the most prominent evidence being demonstrated in the following aspects. First, vegetation decreases building cooling demand by shading and evapotranspiration, thereby avoiding carbon emissions associated with fossil fuel use in energy production (McPherson 1998). Tsoka et al. (2021) report that adding trees can reduce buildings’ cooling energy demand by up to 54%, with foliage density and planting pattern as significate factors of the energy-saving efficacy. Green roofs generally reduce CO_2_ emissions ranging between 1.703 and 1.889 kg/m^2^/year (Kuronuma et al. 2018), but the site selection of green building, as well as facilities accessibility, surrounding environments and residents’ behavior account for 11–50% of the variation in green buildings’ energy preservation (Gill et al. 2010; Gou and Lau 2014). Second, micro-scale features in streets, such as amenities and esthetics, affect human perceptions and can encourage people to engage in more walking, biking, and other pro-environment behaviors (Smardon 1988; Sarkar et al. 2015). Streetscape factors significantly contribute to explaining walking mode choice as well as the associated reduction of automobile carbon emissions. A high odd ratio (1.680–2.070) is found for street-level urban greening to increase walking modal choices (Koo et al 2022). Third, greenbelt policies were found effective in limiting urban growth and promoting a more compact land-use development pattern, preventing land use-associated emissions and carbon stock losses (Han et al. 2022).

A fine-scale analysis of emissions can reveal localized socio-economic drivers of emissions that may not be evident when examining emissions data on a larger scale. This information can be valuable in designing interventions that target specific emission sources, such as reducing emissions from vehicles in urban areas, to achieve maximum emissions mitigation potential. For example, NbS interventions need to be deliberately allocated to areas with major emission sources such as roads with high traffic volume (Gromke and Blocken 2015), buildings with high cooling and heating demand (Xie et al. 2017), and natural carbon stocks near urban development (Ferreira et al. 2018). Similarly, although direct emission mitigation implementations, such as carbon sequestration, can be effective in any location, their benefits can be increased substantially by an optimal land management (Sha et al. 2022). For instance, by coupling eco-environmental measures and relocating significant industrial emission sources, there is a noticeable trend of improvement in removing industrial emissions (Song et al. 2023).

### Challenges to systematically scaling up NbS

There have been recent attempts by many cities to scale up NbS through systematic design, in the hope of acquiring accrued benefits when a number of projects are implemented. Such projects may include developing new NbS and extending or linking existing NbS. Implementing NbS at a large scale requires coordination of multiple sectors, from energy and transport to land-use planning and health (Raymond et al. 2017).

Many projects have demonstrated the potential for moving from siting NbS at single locations to city- or regional-scale deployment and the difficulties in doing so, e.g., the regulatory framework, business models, innovative governance models, and social acceptance that need to be in place before expanding network and knowledge beyond single-case demonstrations (Nesshöver et al. 2017; Bradfer-Lawrence et al. 2021; Cortinovis et al. 2022). Fastenrath et al. (2020) concluded that scaling up of NbS requires interdisciplinary expertise to address ecological, institutional, and socio-cultural challenges. In a participatory analysis of six Swedish municipalities, Wamsler et al. (2020) identified science–policy integration as a key strategy for the success of NbS implementation from a city-to-city learning lab of urban development projects.

In spatial terms, implementing NbS at a larger scale requires a better understanding of the options that fit local environments. The location and size of each NbS affect the benefits it can produce (Cortinovis and Geneletti 2018; Andersson et al. 2020). For example, planting trees can be more easily integrated into spatial plans in less built-up areas than in the urban core, where the possibilities are constrained by existing land uses and spaces (Pataki et al. 2021). Areas of high priority for stormwater abatement are generally not best suited for maximizing landscape connectivity (Meerow and Newell 2017). A more realistic plan for scaling up NbS includes investigating the areas available for different NbS types, identifying appropriate spatial scales of implementation, and evaluating benefits that can be expected from various approaches (Nesshöver et al. 2017; Bradfer-Lawrence et al. 2021; Cortinovis et al. 2022).

Among previous studies on implementing NbS on a regional scale, Midgley et al. (2021) compiled an inventory of water-related ecological infrastructure intervention projects in South Africa and established a range of typologies with specific benefits for landscape actors; Meerow and Newell (2017) developed a GIS-based approach for systematically prioritizing urban green infrastructure based on a wider range of socio-economic and environmental benefits in a heterogeneous landscape; and Cortinovis et al. (2022) showed that characteristics of urban form (i.e., density, impervious area, land use types) affect the potential and benefits of NbS. However, as spatial resolution is important in terms of estimating benefits, finer-scale analysis in such studies would provide better support for implementation. A spatially detailed, systematic, and replicable strategy for selecting and allocating NbS, and for better communicating their benefits, is still lacking.

### Spatially explicit NbS implementation for carbon emission reduction

Spatial modeling specializes in combining and synthesizing different sources of information to assist local communities, planners, and agencies in identifying “hotspots” associated with infrastructure siting, assessing potential spatial tradeoffs, and ultimately enabling more informed plans based on stakeholder input (Geertman and Stillwell 2003). Modeling the spatial interactions between NbS and various systems (e.g., land use, social, environmental) is key in revealing the drivers, feedback, and interactions of the benefits from the large-scale implementation of NbS (Bierwagen et al. 2010; Goldenberg et al. 2017). A study by Madureira and Andresen (2014) identified spatial priority areas for green infrastructure based on two criteria, namely proximity to public green spaces and the potential to improve local temperature regulation. Norton et al. (2015) developed a multi-scale hierarchical model to prioritize GI placement and type, although they mainly focused on the cooling benefits and did not include weighting protocols. Pan et al. (2020) developed a spatially explicit land use model that couples human processes (socio-economic and land use policies) and ecological processes (GHG emissions associated with human activities that have global climate impacts) to understand GHG emissions associated with urbanization and human-driven land-use changes.

Integrating spatial information with social-technology synergy can facilitate the collaborative process in NbS planning. Sarabi et al. (2022) introduced and tested an NbS planning support system (NbS-PSS) that allows users to interact at different stages of the NbS planning process to ensure the fulfillment of societal needs and equitable distribution of ecosystem services. Venter et al. (2021) created a tool for engaging stakeholders in spatial prioritization of green roof retrofitting in Oslo, Norway, and found high spatial correlation in ecosystem services deficits.

There are two key limitations to the current approach of spatially explicit and systematic PSS models: (1) Most urban NbS assessments focus on a limited set of choices, most often street trees or green infrastructure, while little is known about how to select the most effective NbS in different urban contexts; and (2) most models identify neighborhoods for prioritized NbS intervention, while site and solution suitability assessment is not incorporated. To overcome these limitations, this paper presents a modeling framework that integrates the prioritization of NbS measures the and assessment of site suitability in various urban contexts. The framework is sufficiently flexible and replicable to be adopted by cities with access to different types of data.

## Materials and methods

### Overview of methodology

**Fig. 1:**
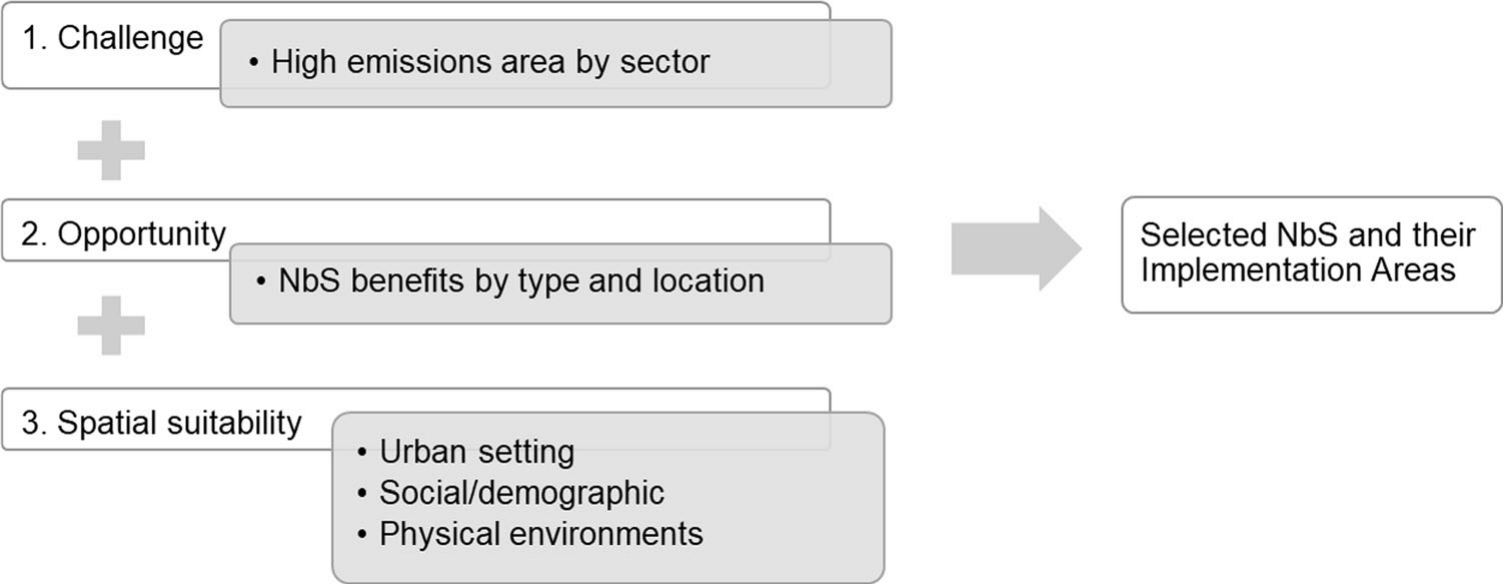
Model framework for spatially explicit deployment of nature-based solutions (NbS)

The construction of a spatially explicit model to identify demands, locations, and types of NbS interventions involved three main steps. First, we studied disaggregated carbon emission maps in the global database for three different sectors (residential, road transportation, industrial), to identify neighborhoods that are likely to cause high levels of carbon emissions and are thus mitigation priority areas. Second, we reviewed the available literature on NbS to compile and assess evidence on the effects of different NbS approaches in offsetting carbon emissions and appropriate siting options. In this step, a set of solutions for the case study area was identified. Third, we spatially deployed NbS interventions based on their expected effects and fine-scale spatial suitability. Carbon reduction benefits are an important criterion when NbS are implemented at a larger scale, but spatial variations in emissions exposure and local conditions can help to identify smaller-scale mitigation priorities. A flowchart of the model is presented in Fig. 1.

### Step 1: Spatially-explicit identification of carbon emission sources

Allocating NbS implementations to intervene with human behaviors and socio-economic dimensions requires a more accurate understanding of the locations and levels of major urban carbon emission sources. Fine-scale emissions analysis can help identify localized socio-economic drivers of emissions, which may not be apparent when looking at emissions data at a larger scale. In order to reveal carbon emission ‘hotspots’ that require policy attention at the local level, we applied a systematic approach to identify carbon emission sources at a 30 × 30 m scale, which is the finest resolution available from land cover maps, from established spatial carbon emission data.

#### Transportation emissions

The literature suggests a positive correlation between pollution concentrations and road traffic (Reynolds and Broderick 2000), and annual average daily traffic (AADT) on road segments (Fu et al. 2017). As traffic volume data at the scale of Stockholm County are not available, we used road classification as a proxy for volume of traffic on its roads. In general, roads of higher classes are designed for higher traffic volume and higher speed, which result in higher emissions production and concentration. In this study, we first used binary dasymetric mapping (Mennis and Hultgren 2006) and set road = 1 and non-road = 0 to identify emissions to linear sources (road), and then adjusted the emission sources in each road segment cell by road class and population density:

1 $$E_{t} = \mathop \sum \limits_{s = 1}^{S} \frac{{C_{t} \times E_{s} }}{{C_{s} }}$$

$E_{t}$: emission source level in target (small) zone t; $E_{s}$: emissions in origin (large) zone s; $C_{t}$: count of road cells in target zone t. $C_{t}$ = 1 in our case, as the target zone is the smallest scale (30 × 30 m) to which data were disaggregated; $C_{s}$: count of road cells in source zone s. This includes motorways, primary roads, and secondary roads. We calculated the value using zonal statistics in ArcGIS:

2 $$e_{t} = E_{t} \times W_{{{\text{class}}}} \times W_{{{\text{pop}}}}$$

$e_{t} :$ transportation emission in kt CO_2_ eq per cell; $W_{{{\text{class}}}}$: weight of road classes. We used a standardized posted speed on each road segment as a proxy and normalized the values to 0–1; $W_{{{\text{pop}}}}$: weight of population density, normalized to 0–1.

#### Residential emissions

We attempted to construct a relationship between residential carbon emissions (*e*_*r*_) and population and building density. As percentage of urbanized area is the main driver of carbon emissions (Vaccari et al. 2013), as model inputs we used the population per cell from EuroStat and the urban fabric density classifications (11 100–11 300) in the Urban Atlas land use database (European Commission 2017):

3 $$e_{r} = f\left( {Den_{{{\text{pop}}}} , Den_{{{\text{building}}}} } \right)$$

$e_{r}$: residential emission in kt _CO2_ eq per cell; $Den_{{{\text{pop}}}}$: population per cell; $Den_{{{\text{building}}}}$: building density per cell, normalized to 0–1.

We used a generic function (*f*() in Eq. 3) instead of a combination of linear parameters because the relationship between urbanization and carbon emission level is often non-linear (Hong 2017; Ahmed et al. 2019). We applied a random forest algorithm to predict emission levels of sources using population counts and building density as fine-scale covariates. Similar approaches have been used by Sorichetta et al. (2015) and Stevens et al. (2015) for predicting population density from aggregated counts to small-area counts. In our model, we used 70% observations as training data and 30% as test data. The model attempts to use 500 trees and the square root of the number of columns at each split as the default parameters. We looked for a better-performing choice of parameters by using a grid search to adjust the number of trees and the number of features with the goal of reducing the root mean square error (RMSE).

#### Industrial emissions

As in the method used for transport data, we applied a binary dasymetric mapping model (Mennis and Hultgren 2006) to identify emission sources from industrial and commercial complexes. To represent the industrial category, we used the land cover type 12 100 in the Urban Atlas land use database, which contains sites of industrial activities, major commercial sites, energy plants, sewage treatment plants, public, military, and private units, etc.

4 $$e_{t} = \mathop \sum \limits_{s = 1}^{S} \frac{{C_{t} \times E_{s} }}{{C_{s} }}$$

$e_{t}$: emissions in target (small) zone t; $E_{s}$: emissions in origin (large) zone s; $C_{t}$: count of industry land use cells in target zone t. $C_{t}$ = 1 in our case, as the target zone was the smallest scale (30 × 30 m) to which data were disaggregated; $C_{s}$: count of industry land use cells at source zone s. We calculated the value using zonal statistics in ArcGIS.

### Step 2: Selection of suitable NBS for direct carbon sequestration and indirect carbon mitigation based on a meta-analysis

Progress in benchmarking the carbon offsetting efforts of NbS is typically constrained by insufficient synthesis of results. To better communicate the effect of NbS, we integrated the findings in existing studies that acknowledge the levels of benefits of different types of NbS toward carbon neutrality.

We applied the qualitative meta-summary techniques proposed by Sandelowski and Barroso (2007) to summarize the mechanisms proposed in the literature. Meta-summary techniques were particularly useful for our purposes, as they synthesize a combination of qualitative and quantitative research findings. Researchers have approached the subject of carbon emission mitigation through a variety of analytical methods that include statistical modeling, simulation, case studies, surveys, and historical data analysis. We used meta-analysis to (1) extract relevant statements on findings from each article; (2) reduce these statements to abstract findings that included the direction and intensity of carbon mitigation effect and the local social and economic conditions in which these methods are applied; and (3) thematize and categorize findings into key NbS strategies that could be used in our subsequent analysis.

To begin, we performed a literature search in Web of Science database on July 16, 2022, using multiple search queries combining keywords associated with NbS and carbon emission issues (see Table 1). After exploring titles, abstracts, and keywords, we limited the search to the document type “articles”, published between 2010 and 2020, and written in English.

**Table 1:** Combination of keywords used to design the search queries

NbS keywords	Linking boolean	Carbon emission keywords
Nature-based solutions OR NbS;	AND	Carbon emissions;
Green roofs;		Climate change;
Urban parks OR public parks;		Carbon sequestration
Street trees;		
Green infrastructure OR GI;		
Preserved habitats;		
Urban agriculture		

From the list of retrieved papers (578 articles), we used different inclusion (IC) and exclusion (EC) criteria (PRISMA, 2020) to exclude studies not relevant to answer the research questions. Articles were excluded after title and abstract screening.

IC1: The paper focuses on the pathways toward zero carbon emissions.

EC1: The paper focuses on estimating the gross volumes of carbon emissions, rather than identifying the mechanisms or pathways toward carbon neutrality.

IC2: The article assesses or quantifies the efficacy of carbon emission reduction strategies.

EC2: The article proposes policy suggestions, but does not examine the quantitative relationship between carbon reduction measures and effects.

IC3: The article investigates carbon mitigation strategies that can be identified as NbS.

EC3: The article investigates strategies that do not fall into NbS categories (e.g., non-fossil fuel energy sources, low-carbon subsidy policies, etc.).

IC4: The article produces transferable metrics (such as elasticity, percentage change, value per unit) that could be applied to other places.

EC4: The article produces case-specific numerical values (such as total carbon emission reduction) that cannot be used to evaluate carbon reduction effects in other places.

The full text of the remaining 54 articles was reviewed against the research questions. In this selection phase, we looked for NbS interventions that involve direct mitigation (e.g., carbon sink) or indirect mitigation (e.g., interventions that could influence human behavior toward low-carbon travel), aiming to include cases representing different NbS approaches to the greatest extent possible. We also intentionally covered research conducted in multiple countries and regions of the world and included a variety of study designs. In total, 20 articles were included in the final review. Table 2 summarizes the basic characteristics of these 20 articles, which focused on cases in 10 countries, including and not restricted to Asian countries (China: *n* = 5, Japan: *n* = 1, South Korea: *n* = 2), USA (*n* = 3), Canada (*n* = 2), European countries (Spain: *n* = 1, Italy: *n* = 2, U.K.: *n* = 1). Fifteen of the 20 articles adopted a case study design where methods included primary data analysis (e.g., field survey) and secondary data analysis (e.g., GIS). The remainder (*n* = 5) conducted energy model simulations or applied cross-sectional analyses based on statistical regression models.

**Table 2:** Basic characteristics of the articles (*n* = 20) included in the review

	Study	Country	Cities	Study design	Study period
1	Anderson and Gough (2020)	Canada	Ontario	Field survey	2017
2	Chen (2015)	China	Multiple Chinese cities	Case study	2010
3	Zhao et al. (2010)	China	Hangzhou	Case study	2000–2002
4	De la Sota et al. (2019)	Spain	Lugo	Case study	40-year horizon
5	Teo et al. (2021)	Global cities		Modeling	2015
6	McPherson et al. (2011)	USA	Los Angeles,	Case study	2007–2010
7	Cai et al. (2019)	China	Wuxi	Experiment	2017–2018
8	Ismail et al. (2012)	Malaysia	Teluk Bahang	Experiment	2008
9	Kuronuma et al. (2018)	Japan		Modeling	45-year horizon
10	Russo et al. (2015)	Italy	Bolzano	Case study	2011
11	Tang et al. (2016)	China	Beijing	Case study	2012–2014
12	Jo et al. (2020)	South Korea	Seoul, Daejeon, Daegu, Chuncheon, Suncheon	Case study	2012–2019
13	Sarkar et al. (2015)	U.K	London	Survey	2005–2010
14	Lindsay et al. (2011)	New Zealand		Case study	2003–2006
15	Vaccari et al. (2013)	Italy	Florence	Case study	2006–2011
16	Escobedo et al. (2010)	USA	Gainesville and Miami-Dade	Modeling	2008
17	Jo et al. (2019)	South Korea	Seoul	Case study	2017
18	Ye et al. (2015)	China	Xiamen	Case study	2009
19	Tomalty (2012)	Canada	Ontario	Case study	2011–2031
20	Han et al. (2022)	USA	Six metropolitan counties	Case study	2006–2016

The following information was extracted from the selected papers:

- Statements indicating the relationship between studied NbS and carbon reduction.
- The approach and indicators used to describe the impact of NbS on carbon emissions.
- Location- or environment-related variables for NbS design.

Our goal in this step was to characterize the functionality of each candidate NbS in its applied urban setting. The result was a matrix-like multi-dimensional overview of the prioritization of different types of NbS approaches based on the spatial characteristics of the study area.

### Step 3: Identify priority areas and NbS intervention types

We combined the high-emission maps produced in the first step and the NbS candidates derived from the second step to explore the question of where each type of NbS can be implemented. We characterized land surfaces across the area based on two groups of indicators: *challenges* and *spatial suitability*, where challenges refer to emissions that every location is dealing with, while *spatial suitability* includes indicators assessing the location’s land use and physical conditions for implementing certain types of NbS. Table 3 lists the spatial data used for the challenges and spatial suitability indicators. A similar classification of indicators is proposed by Kuller et al. (2019) and Sarabi et al. (2022).

**Table 3:** Challenges and spatial suitability indicators

	Indicators	Datasets	Data source
Challenges			
Emissions	CO_2_ emissions by sector	Emissions	Global Carbon Grid (2019)
Spatial suitability			
Urban setting	Population density	Census data	EuroStat (2018)
	Building density	Urban fabric density classifications	Urban Atlas (2018)
	Existing development	Land use data	Urban Atlas (2018)
Physical requirements	Roads	Street network	Tillväxtoch regionplaneförvaltningen (TRF), 2017
	Buildings	Building footprints	

We considered each NbS candidate separately. First, we produced spatial suitability maps by masking out locations where the selected NbS would not be applicable. For example, if the selected NbS was green buildings, only the building footprint area was included in the analysis. Second, we combined the challenges and spatial suitability maps to generate neighborhoods for prioritizing the selected NbS. There are several methods for combining criteria in site selection problems, such as weighted linear combination (WLC) (Malczewski 2006), and the analytic hierarchy process (AHP) method (Parry et al. 2018). In this study, we chose thresholds for high percentile values to identify locations, where implementation was most effective in each map and combined the identified locations with equal weights. Ideally, an inclusive social-technological modeling approach would provide the option for stakeholders to define thresholds (i.e., how large an implementation area should be considered) and choose weights for indicators from both the opportunity and challenge sides.

### Case study site and data sources

**Fig. 2:**
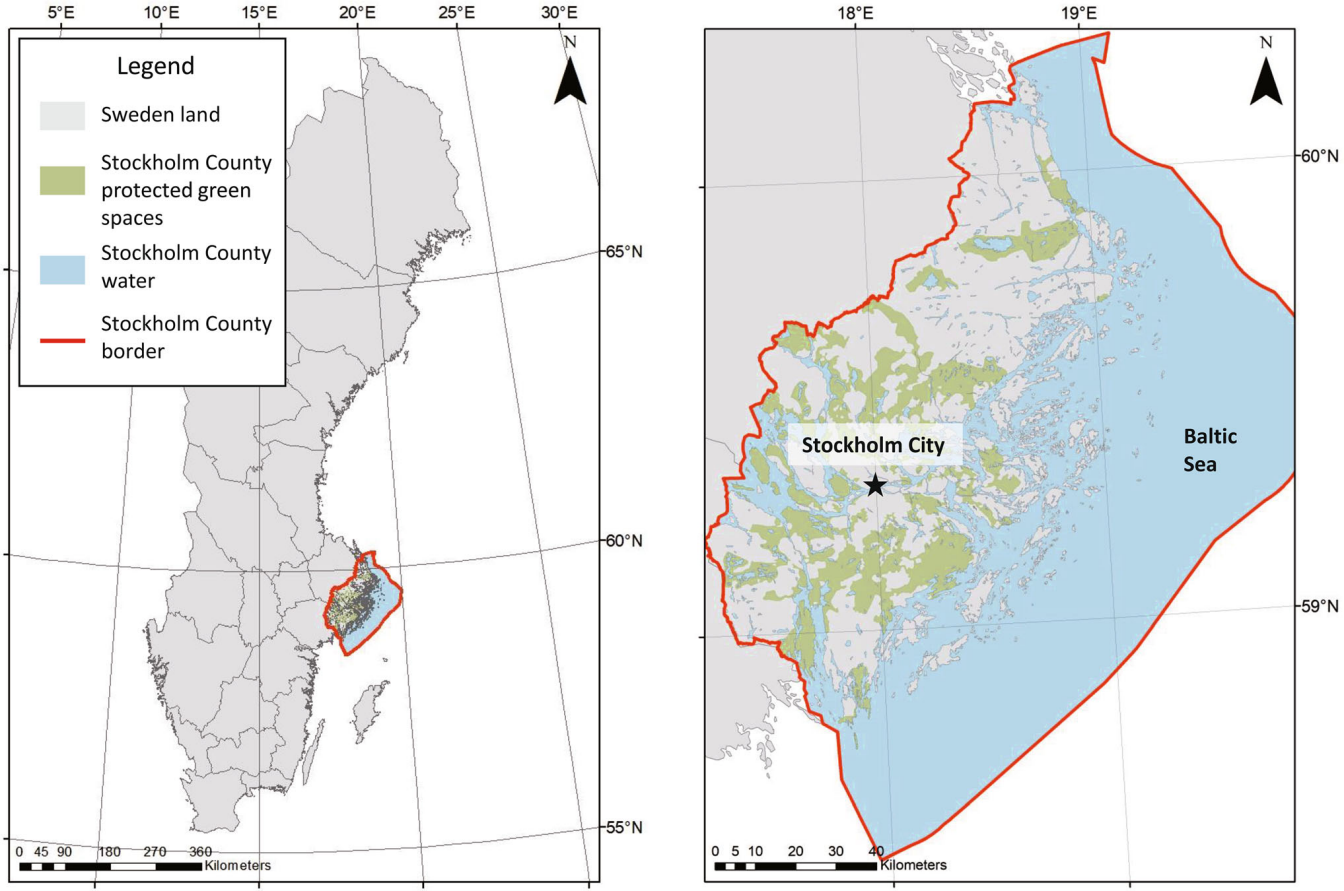
Maps showing (left) the location of the case study area in south-east Sweden and (right) land uses in Stockholm County and location of Stockholm city

Stockholm is the political capital and commercial center of Sweden. In 2020, the estimated population of Stockholm County was 2.3 million and the population within city boundaries was 975 000. The region is expected to continue to grow, with an estimated population of 3.5 million living in Stockholm County by 2050 (TRF 2017). In order to provide infrastructure for this growth and ensure a pleasant city environment, city and county development plans emphasize increasing sustainability through protecting green and blue areas, supporting biodiversity, and improving nature recreation opportunities for residents. The climate policy framework’s long-term climate goal is that by 2045 at the latest, Sweden will have zero net emissions of greenhouse gases to the atmosphere, with negative emissions thereafter. The location and land uses of the study site are shown in Fig. 2.

In our analysis, we used Global Carbon Grid data (Qin et al. 2022) from the Global Infrastructure Emissions Database (http://gidmodel.org.cn/). The Global Carbon Grid provides 0.1° × 0.1° CO_2_ emission maps (year 2019) for six source sectors (power, industry, residential, transport, shipping, and aviation), built upon a framework that integrates multiple data flows, including point sources, country-level sectoral activities and emissions, and transport emissions and distributions. All of these are updated on an annual basis to provide the most up-to-date global emission maps. The location-based estimates lay the foundation for building accurate high-resolution emission maps.

## Results

### Spatial distribution of major carbon emission sources by sector

**Fig. 3:**
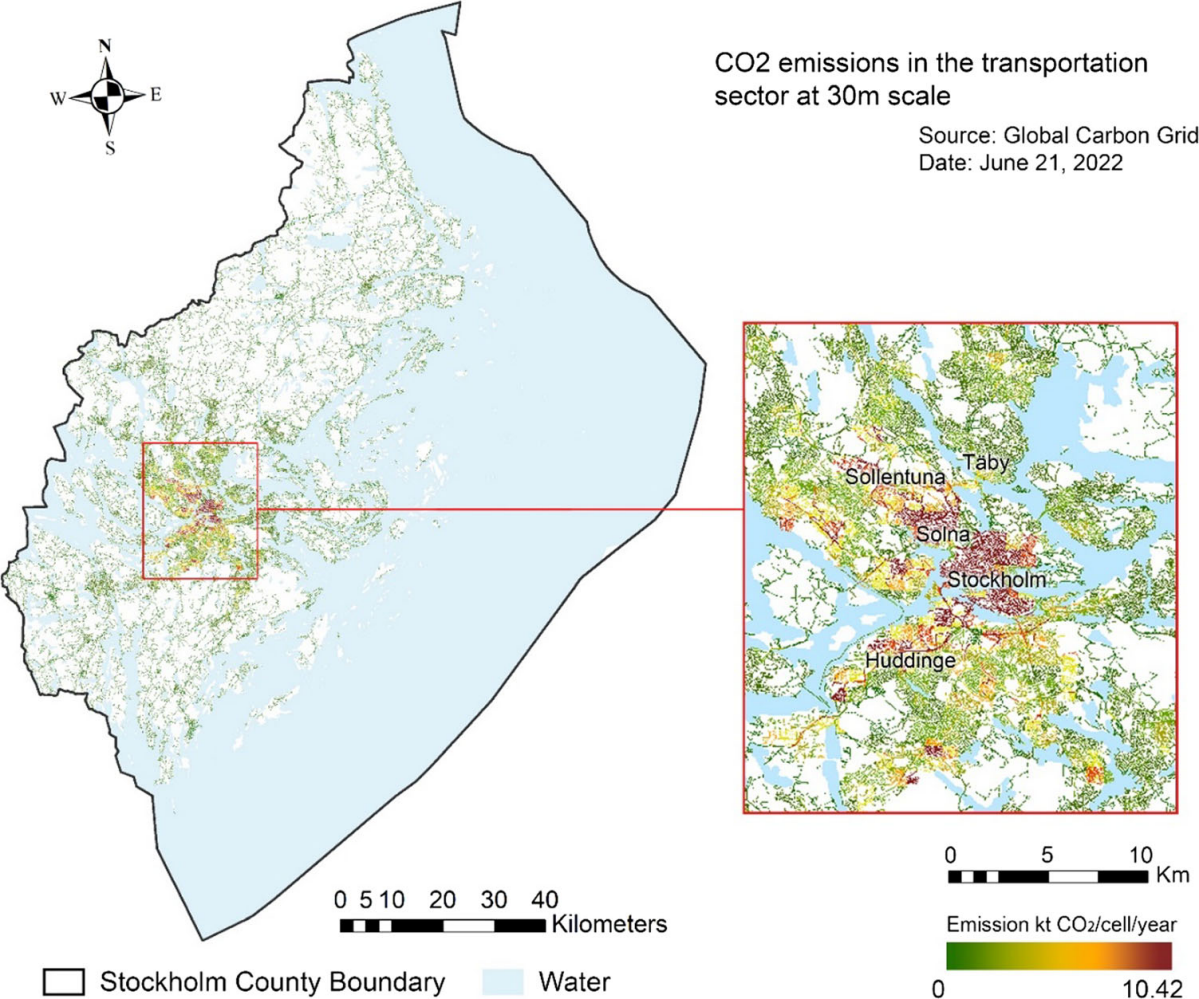
Map of Stockholm County showing the intensity of annual road transportation carbon emissions

By identifying CO_2_ emission sources on a 30 × 30 m scale, we first identified major transportation emission sources in the study region (Fig. 3). Most of these major emission sources are in the existing urban center of Stockholm city and in Solna, due in part to high road density and more human mobility and activities. Medium-to-high emission areas occur around major transportation corridors extending from the urban center toward emerging sub-centers, e.g., in Huddinge, Sundbyberg, Täby, and the Arlanda Airport region. High-travel speeds along highways can be a major contributing factor to the major emission sources in these areas. Rural-to-urban development also increases the inter-city car commute, which means more vehicle-kilometers traveled, more transportation energy use, and hence more emissions.

**Fig. 4:**
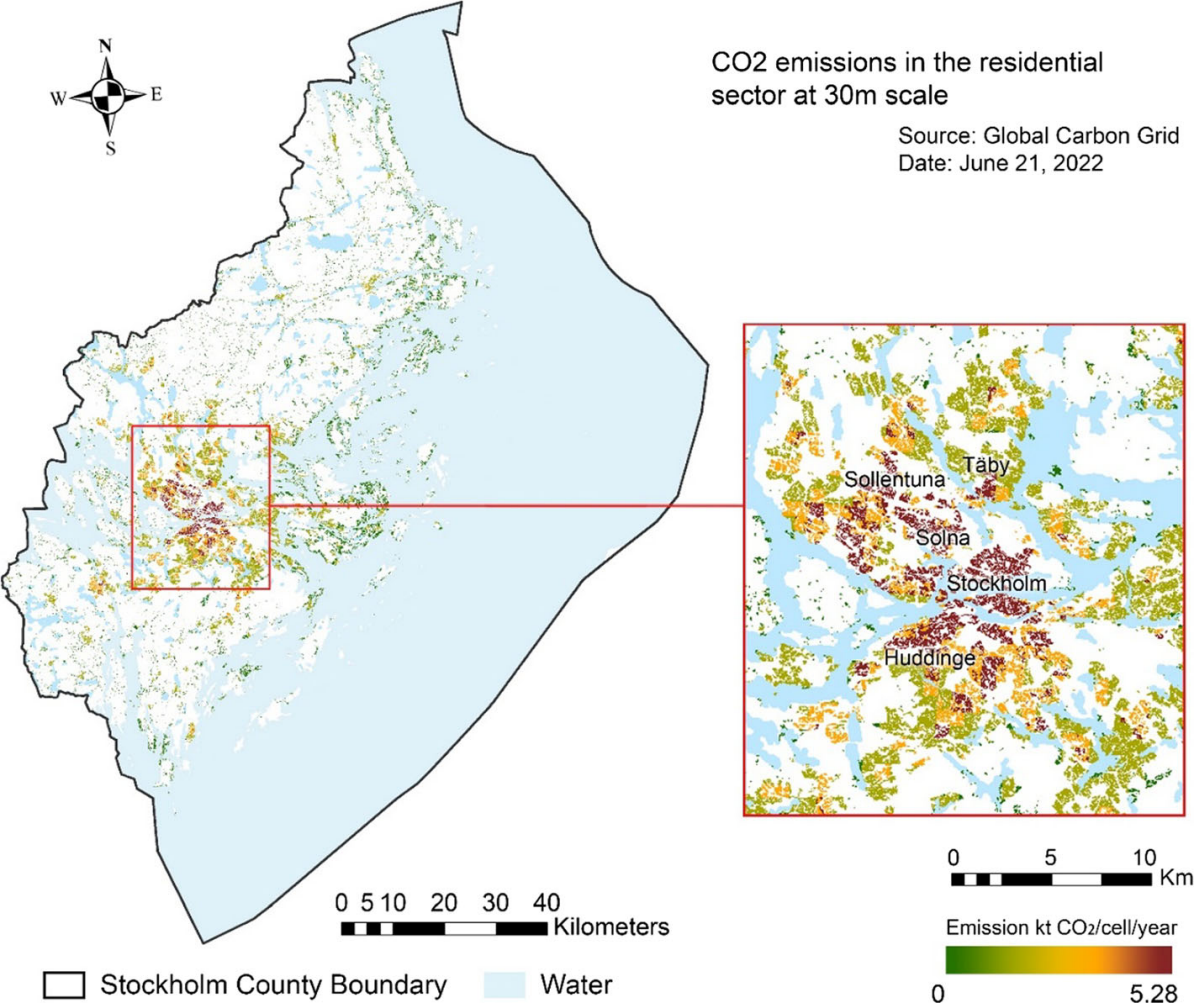
Map of Stockholm County showing the intensity of annual residential carbon emissions

Major residential emission sources are in highly populated areas in Stockholm County, either within the urban center or scattered in peripheral areas than in sparsely populated areas (Fig. 4). This influence is moderated by building density, e.g., single-family developments in north Solna and Sundbyberg, despite lower population density, exhibit almost the same level of emissions as multi-family developments in south Stockholm City. This confirms the previous findings that low-density residential developments are more likely to introduce higher building energy emissions (Pan et al. 2019), a factor that is sometimes overlooked in residential carbon emission forecasts and in urban and environmental planning.

**Fig. 5:**
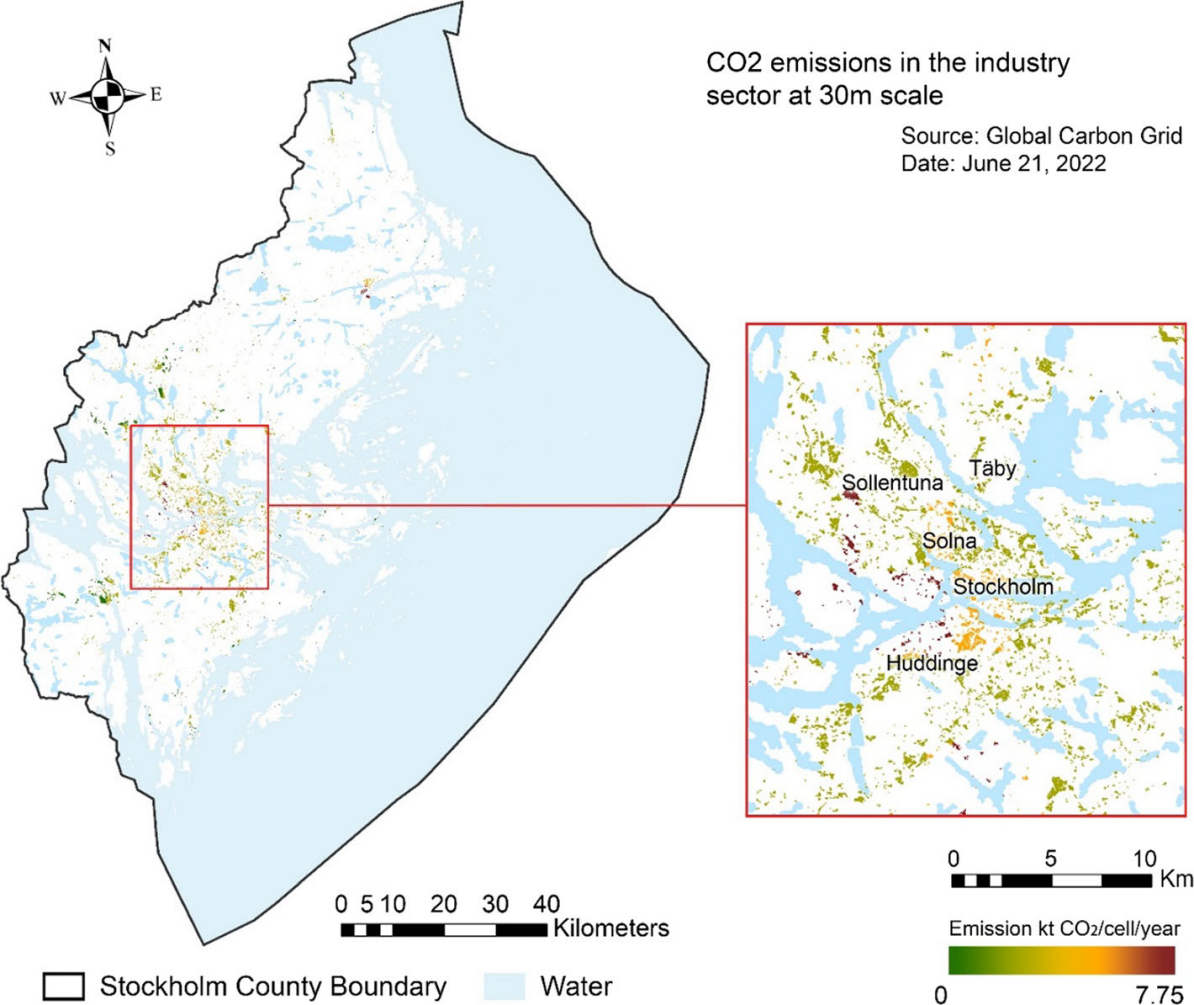
Map of Stockholm County showing the intensity of annual industrial carbon emissions

Industrial and commercial emission sources are usually identified from the combustion of fossil fuels for energy and heat, the use of certain products that contain greenhouse gases, and waste handling (EPA 2020). In our maps for Stockholm County, the distribution of industrial and commercial emission sources showed less obvious patterns associated with urban development and was instead influenced by major energy consumers. Sites of construction companies, building materials suppliers, the automobile industry, and recycling centers displayed higher emissions, even in peripheral or rural areas of the county (Fig. 5).

### Scope for NbS to reduce urban carbon emissions

The mechanisms for achieving zero carbon emissions proposed in our reviewed articles fell within several key NbS strategies. We selected the following five most common types of NbS implemented in urban areas for further study: (1) green and blue infrastructure (GBI), (2) green buildings, (3) street trees, (4) urban green areas, and (5) greenbelt.

The objective of this part of the analysis was to analyze the efficacy of using NbS to mitigate carbon emissions. Developing an efficacy standard is difficult due to the vast differences in green area and structure, plant species, climate conditions, land use, and methodologies used in different studies (Aguaron and McPherson 2012; Strohbach and Haase 2012; Weissert et al. 2014). However, several patterns emerged from our literature review.

1. The potential for trees, urban open spaces, and green facades to offset climate change impacts relates not only to the type and scale of these facilities, but also to their location (Zolch et al. 2018; Sarabi et al. 2022). For example, Vaccaci et al. (2013) found that continuous green areas have higher carbon offsetting rates than fragmented vegetation in densely built-up areas, while De la Sota et al. (2019) highlight the accrued benefits of spatially connecting green infrastructures in carbon mitigation and Ye et al. (2015) advocate locating more accessible green spaces and water bodies in residential areas.
2. Some NbS are more effective in mitigating carbon emissions from one sector than another. For example, green roofs can significantly reduce carbon emissions from the residential sector due to saved building energy (Kuronuma et al. 2018; Cai et al. 2019), while their capacity for capturing transportation-related carbon emissions is negligible and less studied.
3. Although direct comparison between different studies can be problematic, relative metrics such as carbon sequestration rate, carbon emission reduction rate, and percentage of carbon offset from total emissions can indicate the range of carbon mitigation capabilities of each NbS.

Based on Escobedo et al. (2010) and Sarabi et al. (2022), we rated the level of impact of NbS on a scale from 1 to 5 to represent the relative efficacy of NbS in carbon emission reduction, where a value of 5 indicates a relatively high potential to address the challenge and a value of 1 indicates low potential (Table 4). As the impact of each type of NbS is influenced by the social and physical conditions under which it is applied, we further characterized these conditions by urban setting, emission type, and study scale in a multi-dimensional assessment. At this point of the analysis, we roughly categorized urban settings as high-density urban core, peri-urban areas, and rural areas. More detailed urban setting characterization was performed in step 3.

**Table 4:** Nature-based solution (NbS) strategies for different spatial settings and their carbon emission mitigation effects on different sectors. Scores (1–5) represent implementation priority (1 for the lowest and 5 for the highest)

		GBI	Green buildings	Street trees	Urban green areas	Greenbelts
Urban core	Transportation	1	2	4	1	1
	Residential	1	4	3	5	2
	Industry	2	4	3	4	2
Peri-urban areas	Transportation	4	1	3	1	5
	Residential	3	3	2	3	4
	Industry	2	2	1	3	3
Rural areas	Transportation	4	1	2	1	3
	Residential	4	1	1	2	2
	Industry	3	1	1	2	2

### Priority areas and types of NbS interventions

Challenge and spatial suitability maps were produced in this step for each NbS, using the criteria listed in Table 5. The site selection analysis was performed on 30-m grid cells considering the spatial resolution of the utilized datasets. To facilitate the adoption of the system, we mainly used data that are easily accessible for cities (publicly accessible data from European, national, or municipal databases).

**Table 5:** Threshold values used for prioritizing nature-based solution (NbS) intervention areas

	Green–blue infrastructure	Green buildings	Street trees	Urban green areas	Greenbelts
Emissions (challenge)					
Transportation emissions			Top 50th percentile		
Residential emissions		Top 75th percentile			
Industrial emissions		Top 75th percentile			
Urban setting and demographic characters (spatial suitability)					
Population density			Top 90th percentile	Top 90th percentile	Bottom 25th percentile
Building density		Continuous urban fabric			
Physical environment (spatial suitability)					
Building rooftops		Yes			
Existing land use	Forest and semi-natural areas (shrub and/or herbaceous vegetation, open spaces with little or no vegetation)		Road transportation	Open spaces with little or no vegetation	Preserved natural resources, no growth areas, forests, wetlands

**Fig. 6:**
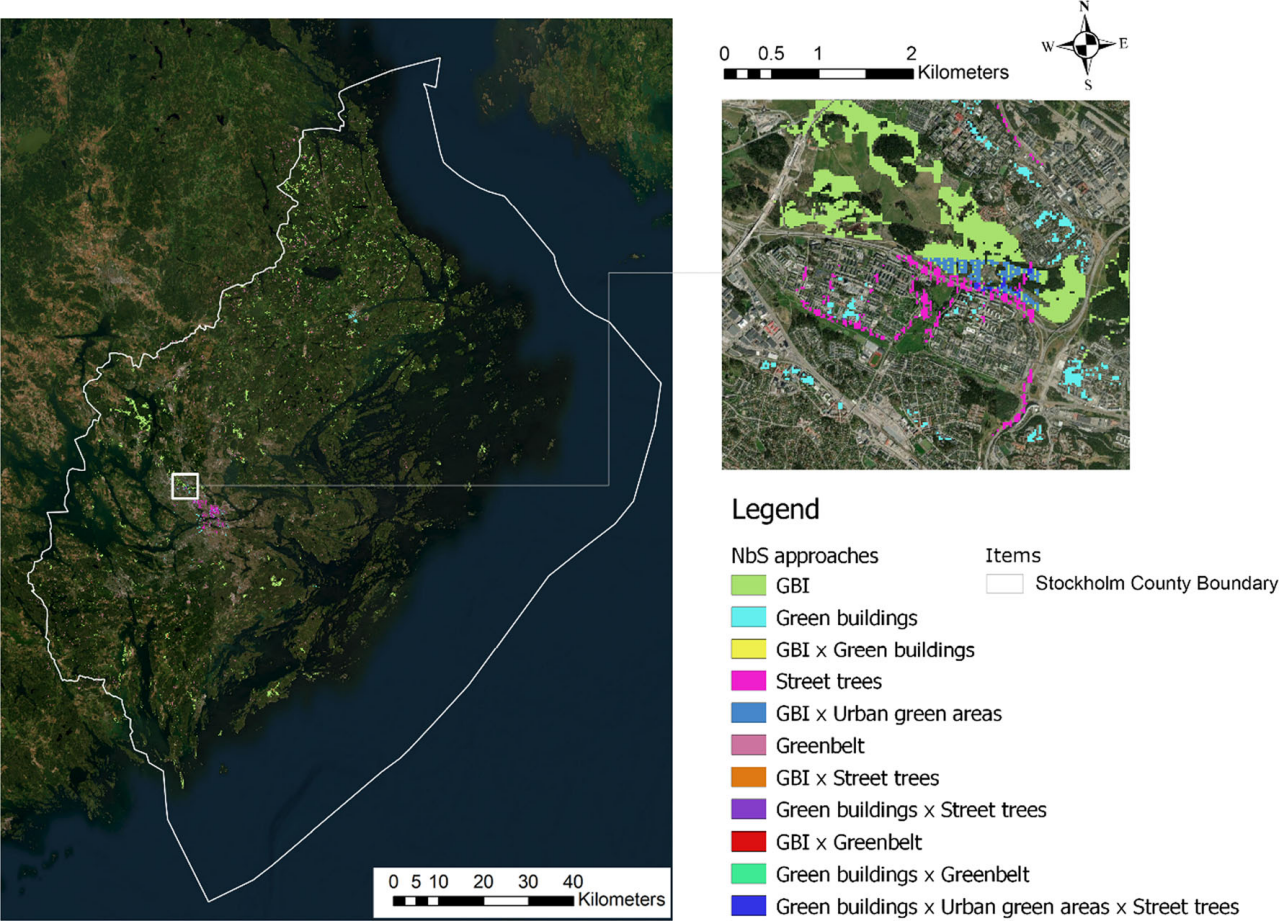
(Left) Areas in Stockholm County prioritized for different nature-based solution (NbS) interventions according to our approach, visualized with a satellite imagery background, and (right) a zoomed-in image of the Sundbyberg area in suburban Stockholm

The final map for NbS prioritized areas revealed several opportunities for the inclusion of NbS in planning and policy to facilitate reductions in overall carbon emissions (Fig. 6, left panel). Increasing street trees emerged as the leading opportunity for the densely developed urban center, while GBI preservation appeared to be an effective approach throughout suburban and rural areas of Stockholm County, due to the city’s forested environment and its high vegetation sequestration potential. Spatial co-location also appeared to be relevant to achieving better cost-effectiveness of urban NbS implementation, e.g., access to green space should be integrated in GBI preservation projects, as some parts of high-quality natural areas can be designed as green spaces to improve green access to residents and workers in the urban fringe.

The large-scale image for suburban Stockholm near Sundbyberg (Fig. 6, right panel) displayed how potential NbS interventions might play out locally. This area mainly includes multi-family residential use and commercial use where green buildings can be expected to achieve high environmental and energy returns. We also identified concentrated areas for street trees, due to the potential for improving the gray infrastructure environment in this medium-to-high density area. GBI interventions should primarily be applied to areas around natural reserves, to prevent future development encroaching into natural environments.

## Discussion

Our results have three major takeaways for practice and policy-making. The first concerns the *spatial distribution of proposed NbS interventions*. While NbS can be deployed throughout urban and rural areas, prioritization of NbS should cater to the specific location, e.g., in urban centers, the most welcome and efficient measures combine artificial and natural green amenities, which includes improving access to green spaces and street trees simultaneously (Samuelsson 2021). Implementation of these measures requires policy on urban natural amenities, but also social policies such as targeting disadvantaged areas, identifying disparities, removing physical barriers (gated green spaces), partnership, and participation (Gupta et al. 2016; Biernacka and Kronenberg 2018; Kronenberg et al. 2020). For rural and suburban areas, the most important NbS measures relate to preserving and utilizing natural areas, which include greenbelt, grass strips, and other forms of green infrastructure. Natural features, including urban forests, transitional ecosystems, and local natural reserves, can provide ecosystem services for urban areas, save infrastructure costs, and prevent urban sprawl (Gavrilidis et al. 2019).

The second practical takeaway from our findings is that *NbS measures can benefit more than one carbon emissions sector (transportation, residential, industry) and generate co-benefits at places where multiple NbS interventions are prioritized*. Our spatially detailed analysis revealed the potential for the co-existence of multiple NbS approaches. Urban centers and sub-centers with high road density and high population density can benefit from approaches ranging from green infrastructures to street environments, quality-of-life amenities, accessible recreational opportunities, and green roofs. Implementation of these NbS interventions would generate co-benefits for the residential, transportation, and industrial sectors.

The third practical takeaway concerns *the behavioral influences of implementing NbS*. In this study, we assessed both direct and indirect pathways for NbS to achieve carbon savings goals. Direct effects such as preserving green spaces are widely reported in the literature (Li and Wang 2021; Page et al. 2021; Ramchunder and Ziegler 2021), but using NbS as a potential tool for nudging and influencing human behaviors is a significant, but often overlooked, opportunity in policy-making to achieve carbon neutrality (Linder et al. 2022). For example, improving streetscapes in the urban center not only contributes to transportation emission reduction but is also beneficial for fostering a walking and biking culture and building an environment-aware civil society (Ewing et al. 2015; Cain et al. 2017; Liu et al. 2020). The socio-psychological aspects of change were highlighted by Seyfang and Haxeltine (2012), aiming at a shift in the behavioral norms and societal shifts in values and beliefs (Westley et al. 2011). We thus see an urgent need to include behavioral and resilience building interventions, such as green education, new green jobs, participatory place-based learning and experimentation, and inviting civic organizations into NbS management, in a wider urban NbS toolbox to achieve urban carbon neutrality.

NbS has many other benefits beyond climate actions, such as enhancing habitat quality, promoting recreational opportunities and human health, increasing equitable access to amenities, and creating jobs in the green sector (Raymond et al. 2017; Giordano et al. 2020; Ruangpan et al. 2021). Due to space restrictions, we focused our investigation on carbon emission effects, but future studies should acknowledge the multifunctionality and co-benefits of NbS in all environmental and socio-ecological dimensions.

## Conclusions

This paper presents a novel systematic framework for identifying the need for NbS interventions at the local scale and for prioritizing sites and strategies for NbS interventions. By moving from the global to the neighborhood level, we identified NbS strategies that can maximize carbon emissions saving potential given local conditions, and sites where they should be implemented. In our case study, the city center and surrounding areas of Stockholm were shown to require different types of NbS to maximize the carbon-saving benefits needed to transform Stockholm into a carbon-neutral city. In the urban center, the most welcome and efficient measures combine improving access to green spaces and streetscapes. In rural and suburban areas, the most important measures related to preserving and utilizing natural areas, which include greenbelt and green infrastructure. Natural features, including urban forests, transitional ecosystems, and local nature reserves, can provide ecosystem services to urban areas, saving infrastructure costs and preventing urban sprawl.

The framework developed in this study can be applied to address a variety of urban sustainability and resilience challenges, such as heat stress, stormwater management, air quality, landscape connectivity, and spatial equity, each of which will require a specific set of mitigating approaches. In a typical application, one could first rank neighborhoods in terms of the level of exposure and/or vulnerability to a set of challenges, then prioritize hazard-mitigating approaches based on their benefits and impacts, and finally, conduct rule-based suitability analysis to overlay social criteria and physical conditions and generate spatially detailed maps. Our proposed method can help planners better select target locations for intended risk/hazard-mitigating interventions.

One methodological limitation of this study was the lack of empirical data and such analysis can be enhanced as further evidence becomes available. Land cover type data from Urban Atlas are useful but could be complemented with additional socio-economic variables to better account for local-scale emissions. Further, our analysis only captured the physical environment where NbS can be applied, so in future studies implementation of NbS should be coordinated with other environmental and social benefits across social groups.

Another limitation is that our study did not focus on iterating solutions for a more collaborative spatial planning process. While our methods provide spatially detailed solutions for ensuring that NbS actions align with existing and/or proposed urban planning strategies and governance processes, further studies can integrate the analysis into a collaborative planning process to facilitate greater stakeholder inputs in terms of identifying suitable intervention areas and solutions. Practitioners need to integrate diverse types and systems of knowledge and values for NbS design and implementation to ensure that plans are socially comprehensible and acceptable.
